# Production losses due to mortality associated with modifiable health risk factors in Poland

**DOI:** 10.1007/s10198-021-01345-6

**Published:** 2021-07-08

**Authors:** Błażej Łyszczarz, Karolina Sowa

**Affiliations:** 1 Department of Health Economics, Nicolaus Copernicus University in Toruń, Toruń, Poland; 2 Department of Analyses and Strategies, Ministry of Health, Warsaw, Poland

**Keywords:** Risk factors, Production losses, Indirect cost, Poland, Global Burden of Disease, I10, I18

## Abstract

**Background:**

Epidemiological burden of modifiable mortality risk factors is recognized in literature; however, less is known on the economic losses due to a range of such risks.

**Aim:**

To estimate production losses (indirect cost) of mortality associated with risk factors as classified in Global Burden of Disease 2019 Study in Poland in years 2000, 2010, and 2017.

**Methods:**

We relied on the human capital method and societal perspective and used sex-, age-, region-, and risk-specific data on mortality due to modifiable risk factors and a set of socio-economic measures.

**Results:**

The production losses due to mortality attributable to all investigated risk factors accounted for 19.6–21.0 billion PLN (Polish zloty; 2017 exchange rate: 1€ = 4.26 PLN) and 1.44–2.45% of gross domestic product, depending on year. Behavioural factors were the most important contributor to overall burden (16.7–18.2 billion PLN), followed by metabolic factors (6.8–7.6 billion PLN) and environmental and occupational factors (3.0–3.5 billion PLN). Of disaggregated risks, alcohol and tobacco, high systolic blood pressure, and dietary risks proved to lead to the highest losses. Cost per death was greatest for child and maternal malnutrition, followed by intimate partner violence and childhood sexual abuse and bullying. Moreover, a notable regional variation of indirect cost was identified with losses ranging from 1.21 to 1.81% of regional gross domestic product in 2017.

**Conclusion:**

Our findings provide economically hierarchised list of modifiable risk factors and they contribute to inform policy-makers in prioritizing programmes to improve health.

**Supplementary Information:**

The online version contains supplementary material available at 10.1007/s10198-021-01345-6.

## Introduction

Economic burden of health problems and diseases is one of the extensively researched areas in public health [1, 2] and this issue has a vital role in health policy decision-making, including public financing of health interventions and prioritizing diseases’ treatment [3, 4]. A majority of research in this area focuses on specific diseases and less evidence is available on the economic consequences of health risks. Although there are studies concerned with cost of alcohol [5], tobacco and drug use [6], dietary risks [7], physical inactivity [8], air pollution [9, 10], and other health risks, the evidence on the economic burden of a comprehensive range of risk factors is scarce. The only study concerned with cost of an exhaustive set of modifiable health risks is an American attribution analysis investigating health care spending associated with 84 risk factors [11]. According to this study, spending on treating diseases resultant from these risks accounted for US$730.4 billion, 27% of total health expenditure in the US in 2016. Another study examining economic aspects of multiple health risks scrutinized the effect of smoking, binge drinking, physical inactivity, and poor diet on being high-cost users of health care in Canada [12]. Moreover, a recent research assessed future medical cost burden for the European health systems under alternative exposure-to-risks scenarios [13]. However, the evidence on economic burden of numerous health risks is absent in the area of production losses (indirect cost); to the best of our knowledge, none of the studies has investigated this economic aspect for a range of risk factors.

This study aims to fill this gap by applying the data on modifiable risk factors estimated in the Global Burden of Disease, Injuries, and Risk Factors (GBD) Study 2019 [14] to identification of production losses attributable to these risks. We aim to do so by estimating country-level economic burden resulting from production losses attributable to premature deaths due to a range of modifiable risk factors as classified by GBD. For this purpose, we used data regarding risk factors analysed with sub-national (region-level) epidemiological estimates from Poland. This approach allowed to compare the economic burden of behavioural; environmental and occupational; and metabolic factors (Level 1) and twenty Level 2 factors in terms of the premature mortality indirect cost borne by Polish society. Clearly, our analysis does not capture the whole economic burden of risk factors as it does not identify direct cost of treating diseases resultant from these risks. Moreover, we limit this study to mortality solely. Although we are aware of potential inclusion of morbidity dimension into our analysis, we argue that the monetization of disability-adjusted life-years (DALYs) (which is one of the measures reported in GBD) for the purpose of production losses estimation is not a common practice. Such an analysis shall follow, once more consensus is achieved regarding methodological approach to such valuation [15, 16]. Additionally, there is a strong correlation between mortality and DALYs measures (correlation coefficient value of 95.4% for deaths and DALYs in country-level, all ages, both sexes combined Level 2 risks, 2017 data), thus, the analysis of mortality solely does not disturb the structure of losses broken down by risk factor. Furthermore, the cost analyses of mortality as a single cost component are numerous (see, e.g., [17–19]), and therefore, our approach is not distinct from other studies.

Hence, the purpose of this study was to estimate production losses (indirect cost) associated with mortality due to modifiable risk factors (as classified by GBD 2019 Study) in Poland using region-level data for years 2000, 2010, and 2017. Knowledge of production losses attributable to risk factors analysed here is important for prioritizing preventive actions and health promotion. The original contribution of this research is to provide a first estimate of production losses attributable to mortality from a wide range of risk factors. Moreover, this study benefits from sub-national-level data which increases the precision of estimates and provides results for three distant periods allowing to track time trends in economic burden.

## Methods

### General approach

This study used population-level data, societal perspective [20, 21], and human capital method (HCM) [4, 22] to estimate the production losses associated with premature mortality due to Level 0, three Level 1, and twenty Level 2 risk factors (according to GBD) in Poland. We used region-level data (NUTS-2 level according to European Union’s nomenclature of territorial units) and estimated production cost for 3 years—2000, 2010, and 2017.

GBD 2019 is a study which estimated, i.e., mortality for risk factors and combination of risk factors at various geographical units, including countries. It provides a standardised and comprehensive assessment of risk exposure and burden attributable to these risks by following the general framework established for comparative risk assessment. GBD risk factor hierarchy applied in this study used three levels of factors which contribute to mortality and morbidity of populations. Here, we used data on mortality due to risk factors assigned to Level 0 (all risks combined), Level 1 (behavioural; environmental and occupational; and metabolic factors), and Level 2 [air pollution; alcohol use; child and maternal malnutrition; childhood sexual abuse and bullying; dietary risks; drug use; high body-mass index (BMI); high fasting plasma glucose; high LDL cholesterol; high systolic blood pressure; intimate partner violence; kidney disfunction; low bone mineral density; low physical activity; non-optimal temperature; occupational risks; other environmental risks; tobacco; unsafe sex; unsafe water, sanitation and handwashing] of GBD risk classification [14].

Because of the GBD study design, the total cost attributable to a particular group of risk factors (e.g., metabolic factors—Level 1) is not the sum of costs attributable to individual risk factors included in this group (six Level 2 factors included in metabolic factors). This results from the fact that risk factors are investigated individually, while some risks are mitigating factors for other risks also included in the study. As a result of these relationships, attributable losses estimated for a group of factors cannot be aggregated. Instead, the GBD approach uses joint population attributable fractions for each level of the risk hierarchy to avoid double counting that would result from simply summing up attributable fractions for individual risk factors [11].

We investigated losses associated with formal economy solely, because we used the gross domestic product as a productivity measure, no measures of losses due to informal activities as housekeeping, informal care, etc. were included. Premature deaths were defined as those mortality cases that occurred at working age. The use of HCM means that the production cost of mortality was proxied by the discounted value of output that would be produced if those who died prematurely were still alive and working until retirement [23]; we also accounted for labour participation rates. The measure of economic output used was per employee gross domestic product (GDP) adjusted for decreasing marginal productivity (by applying a 0.65 coefficient). We used this adjustment, because a marginal productivity is preferred over an average productivity in production losses estimation [24]. This results from the fact that the production output relies not only on human capital but also other inputs as capital or land. When fewer workers contribute to production process (due to, e.g., premature mortality), the economic output is decreased less than proportionally, and therefore, the GDP lost should not reflect average, but marginal productivity. We proxy marginal productivity with 0.65 coefficient which reflect output elasticity of labour in Cobb–Douglas production function as used in the European context [25].

Each time, region- and sex-specific data were obtainable we used it; otherwise, we relied on country-level figures; detailed information on the level of particular measures used is provided further in this section.

### Data sources

The age-group- (5-year intervals), sex-, and region-specific data on the number of deaths due to Level 0, three Level 1, and twenty Level 2 risk factors were extracted from Poland’s Ministry of Health online platform ‘The Database of Systemic and Implementation Analyses’ [26]. These figures account for joint population attributable fractions as explained above. The region-specific data on social, economic, and population measures used were taken from Local Data Bank of Statistics Poland [27]. The average effective age of retirement [28] and average age of starting first regular job (received from Eurostat on the authors’ request) were only obtainable at country level; moreover, the data on the former were available for each of the 3 years investigated, while for the latter, we relied on uniform figures for the year 2015. For future potential per-worker GDP, we also relied on country-level data [29] as region-level forecasts were not found.

### Estimation strategy

In the first step, the age-, region-, and sex-specific prevalence of deaths (per 100,000 population) associated with each of risk factors at 5-year age intervals (under 5; 5–9; …; 60–64; 65–69 years) was extracted from Ministry of Health database [26] for the years 2000, 2010, and 2017. The product of these figures and population data resulted in a number of deaths for each of risk factors. Furthermore, we assumed even distribution of death cases for particular ages in each age interval. A half-cycle adjustment was applied assuming that all deaths occurred in the middle of the year [30]. We used a measure of years of potential productive life lost (YPPLL) to weight mortality occurring at various ages.

In the second step, we identified the average time a person at each age would work if had not died prematurely. This was done by using country-level data on average age of starting first regular job and average age of exiting the labour market [28] as well as region-level data on employment rate at working age [27]. All of the above were sex-specific figures. Because of uncertainty of future labour market trends and lack of forecasts on this issue, we assumed the above measures to be constant in the following years.

In the third step, the indirect cost of an early death at each working age and separately for men and women was identified by summing discounted (5% discount rate) values of production lost for each year of potential productive life lost. This was done using region-specific per-worker real GDP (2017 being a base year), application of marginal productivity coefficient (0.65), and with the use of predicted growth rates of per-worker GDP in the country [29].

### Sensitivity analysis

A one-way sensitivity analysis of deterministic character was performed to assess the stability of our base scenario (BS) estimates for changes in the model’s parameters. The following scenarios were investigated: Sc_1 (Sc_2)—assumption that all the death cases in a particular 5-year interval occurred at the first (last) year of the interval [e.g., for 10–14 interval, deaths at the age of 10 (14)]. Scenarios Sc_3, Sc_4, and Sc_5 used 0%, 2%, and 3.5% discount rates, respectively, instead of 5% BS rate. In Sc_6 and Sc_7, we tested how the results would be affected by assuming 0% and 2% future economic growth, while scenarios Sc_8 and Sc_9 deviate productivity adjustment coefficient by ± 0.05. Scenarios Sc_10 used country-level instead of region-level data and Sc_11 applied gross value added (GVA) instead of GDP as a productivity measure.

## Results

### Years of potential productive life lost

The total number of years of potential productive life lost (YPPLL) attributable to all risk factors together was 617.9 thousand in 2000 and it decreased to 521.1 thousand in 2010 and 463.8 thousand in 2017 (Table 1). This translated to per 100,000 population values of 1,615 YPPLL in 2000, 1,353 YPPLL in 2010, and 1,207 YPPLL in 2017 (Supplementary file 1). Majority of the YPPLL burden in each of the years was associated with deaths due to behavioural factors with alcohol use (197.8 thousand YPPLL in 2000, 205.2 thousand YPPLL in 2010 and 191.3 thousand YPPLL in 2017), tobacco (228.3 thousand YPPLL in 2000, 160.9 thousand YPPLL in 2010, and 134.7 thousand YPPLL in 2017) and dietary risks (141.1 thousand YPPLL in 2000, 98.5 thousand YPPLL in 2010, and 84.9 thousand YPPLL in 2017) being major contributors. High systolic blood pressure and high BMI both resulted in > 80 thousand YPPLL in 2017 and constituted the most important non-behavioural causes of mortality at working age economic burden. Of the environmental and occupational factors, air pollution resulted in the highest number of YPPLL; however, this burden declined notably during the period investigated, from 88.5 thousand in 2000 to 45.4 thousand in 2017. One of the factors (non-optimal temperature) had ameliorative or preventative effect on mortality and resulted in reduced YPPLL (− 7.8 thousand in 2000 to − 1.0 thousand in 2017) (Table 1). Clearly, the data on rates of YPPLL per 100,000 population reflect the trends and structure described above for the total numbers; yet, for the sake of brevity, it was not presented in the main text and the detailed figures can be found in Supplementary file 1.

**Table 1:** Years of potential productive life lost attributable to modifiable risk factors in Poland in years 2000, 2010, and 2017

	2000			2010			2017		
	Males	Females	Total	Males	Females	Total	Males	Females	Total
Environmental and occupational factors	87,483	22,021	109,504	65,211	14,893	80,104	54,671	11,503	66,174
Air pollution	69,436	19,041	88,477	49,562	12,195	61,757	36,713	8646	45,359
Occupational risks	26,360	2484	28,844	22,426	1986	24,412	20,283	1808	22,091
Other environmental risks	7582	930	8511	5180	643	5823	3794	526	4320
Unsafe water, sanitation, and handwashing	367	182	549	285	126	411	263	100	363
Non-optimal temperature	− 9073	1320	− 7753	− 6459	1270	− 5189	− 2287	1332	− 955
Behavioural factors	440,232	107,524	547,757	378,850	79,248	458,098	335,404	67,239	402,644
Alcohol use	182,413	15,407	197,821	188,452	16,777	205,229	174,821	16,433	191,253
Tobacco	182,417	45,930	228,347	130,893	30,003	160,895	109,700	25,015	134,715
Dietary risks	116,517	24,611	141,128	82,942	15,577	98,519	71,677	13,225	84,902
Drug use	14,051	2178	16,228	15,368	2177	17,545	14,693	2298	16,992
Child and maternal malnutrition	33,510	22,347	55,857	23,343	15,995	39,338	15,811	10,596	26,406
Unsafe sex	1590	15,147	16,737	1705	9344	11,049	1363	7905	9268
Childhood sexual abuse and bullying	5399	206	5604	5625	253	5877	5799	277	6076
Low physical activity	3036	1105	4141	2253	777	3029	2355	747	3102
Intimate partner violence	0	787	787	0	424	424	0	357	357
Metabolic factors	188,379	44,471	232,850	145,669	29,860	175,530	127,247	24,581	151,828
High systolic blood pressure	116,441	23,783	140,224	94,672	16,234	110,907	77,204	11,683	88,888
High body-mass index	90,354	25,365	115,719	72,663	16,868	89,531	66,666	14,509	81,175
High LDL cholesterol	93,691	14,062	107,753	60,471	7804	68,274	48,697	6062	54,759
High fasting plasma glucose	32,530	8975	41,505	31,188	7109	38,296	31,747	6577	38,323
Kidney disfunction	19,554	6560	26,114	13,707	3904	17,611	10,968	2892	13,860
Low bone mineral density	5315	1092	6407	3871	674	4545	3291	575	3866
All risk factors	491,413	126,508	617,920	426,694	94,454	521,148	383,001	80,831	463,831

Considering sex distribution of YPPLL, for the all-cause (Level 0) measure, the share of male YPPLL was 79.5% in 2000 and it increased to 82.6% in 2017. The share of male burden was as much as 95–96% for childhood sexual abuse and bullying, and 91–92% for occupational risks and alcohol use. On the other hand, all the YPPLL resulting from intimate partner violence concerned females and other factors affecting women relatively heavily were unsafe sex (85–90% of total YPPLL) and child and maternal malnutrition (40–41% of total YPPLL).

### Production losses attributable to modifiable risk factors

Total production losses due to premature mortality caused by all risk factors combined in Poland were 19.6 billion PLN in 2000 (from herein, all PLN values are expressed in real terms with 2017 being a base year; average 2017 exchange rate: 1€ = 4.26 PLN; 1US$ = 3.78 PLN), declined to 19.1 billion PLN in 2010, and increased to 21.0 billion PLN in 2017 (Table 2). Behavioural factors were a group of mortality determinants associated with the highest indirect cost—16.7–18.2 billion PLN, depending on year. Metabolic factors’ burden accounted for 6.8–7.6 billion PLN while environmental and occupational risks led to production loss of 3.0–3.5 billion PLN.

**Table 2:** Production losses due to mortality attributable to modifiable risk factors in Poland in years 2000, 2010, and 2017 (thousands of PLN)

	2000			2010			2017		
	Males	Females	Total	Males	Females	Total	Males	Females	Total
Environmental and occupational factors	2,909,779	594,154	3,503,932	2,559,023	479,589	3,038,612	2,618,228	474,338	3,092,566
Air pollution	2,294,254	509,273	2,803,527	1,911,827	385,820	2,297,648	1,742,558	350,216	2,092,775
Occupational risks	864,290	69,962	934,251	823,482	65,887	889,369	906,745	75,476	982,221
Other environmental risks	251,846	26,273	278,120	203,117	22,248	225,366	181,757	22,848	204,605
Unsafe water, sanitation, and handwashing	9731	3468	13,198	8691	2568	11,259	10,675	2556	13,231
Non-optimal temperature	− 268,024	38,833	− 229,191	− 155,798	47,305	− 108,493	− 19,617	61,539	41,922
Behavioural factors	14,475,033	2,841,013	17,316,046	14,247,276	2,456,770	16,704,046	15,492,038	2,672,741	18,164,779
Alcohol use	6,171,865	439,586	6,611,451	7,160,023	564,605	7,724,629	8,131,045	699,692	8,830,737
Tobacco	6,163,564	1,309,881	7,473,445	5,195,927	1,039,196	6,235,123	5,315,870	1,094,033	6,409,903
Dietary risks	3,960,496	699,530	4,660,027	3,288,085	534,709	3,822,794	3,475,824	573,630	4,049,453
Drug use	475,098	62,411	537,509	582,266	73,886	656,152	675,487	98,647	774,134
Child and maternal malnutrition	721,640	411,357	1,132,997	481,425	289,144	770,568	375,430	225,669	601,099
Unsafe sex	54,500	433,670	488,170	64,018	320,606	384,624	61,887	339,190	401,077
Childhood sexual abuse and bullying	184,460	5944	190,404	215,936	8566	224,502	273,517	11,829	285,346
Low physical activity	103,586	31,341	134,927	90,907	27,060	117,968	116,847	33,013	149,860
Intimate partner violence	0	22,365	22,365	0	13,730	13,730	0	14,501	14,501
Metabolic factors	6,384,012	1,248,540	7,632,551	5,762,540	1,017,436	6,779,975	6,161,940	1,060,554	7,222,493
High systolic blood pressure	3,931,879	666,850	4,598,729	3,749,983	557,083	4,307,066	3,743,081	507,722	4,250,804
High body-mass index	3,090,995	722,391	3,813,386	2,896,050	583,282	3,479,333	3,250,608	632,634	3,883,242
High LDL cholesterol	3,194,338	398,541	3,592,879	2,416,924	269,621	2,686,546	2,384,046	265,304	2,649,350
High fasting plasma glucose	1,098,649	252,550	1,351,199	1,232,822	243,245	1,476,067	1,539,205	284,620	1,823,825
Kidney disfunction	650,879	178,742	829,621	527,714	124,545	652,259	518,856	117,327	636,182
Low bone mineral density	185,567	32,055	217,622	154,977	24,108	179,085	160,843	25,947	186,790
All risk factors	16,201,758	3,367,768	19,569,526	16,139,810	2,969,908	19,109,718	17,778,659	3,253,635	21,032,293

Costs are expressed in real values (2017 is a base year)

Alcohol use was a single Level 2 mortality risk factor associated with the highest economic losses and its burden increased from 6.6 billion PLN in 2000 to 7.7 billion PLN in 2010 and 8.8 billion PLN in 2017. The only risk factor of similar economic burden was tobacco; however, in contrast to alcohol use, the magnitude of tobacco indirect cost declined throughout the period. In 2000, tobacco led to losses of 7.5 billion PLN, while this burden decreased by more than a billion PLN in 2010 and 2017. The other behavioural risk factor of notable importance for mortality economic burden were dietary risks (3.8–4.7 billion PLN). Of the metabolic factors, the ones leading to substantial mortality production losses were high systolic blood pressure (4.3–4.6 billion PLN), high BMI (3.5–3.9 billion PLN), high LDL cholesterol (2.6–3.6 billion PLN), and high fasting plasma glucose (1.4–1.8 billion PLN). The two environmental and occupational risk factors which caused relevant mortality cost were air pollution (2.1–2.8 billion PLN) and occupational risks (0.9–1.0 billion PLN). Non-optimal temperature had a cost-saving effect among males leading to a minor benefit of 20–268 million PLN, while in females, it resulted in losses of 39–62 million PLN.

A majority of Level 0 production losses caused by premature mortality (83–85% depending on year) were associated with deaths of males. This share of costs attributable to male deaths was highest (> 90% in each of the years) for occupational risks, alcohol use, and childhood sexual abuse and bullying. On the other hand, the economic burden of mortality resulting from intimate partner violence, unsafe sex, and non-optimal temperature concentrated in female mortality (Table 2).

A more nuanced picture arises from analysing relative measures of premature mortality economic burden by risk factor (Table 3). While by-risk losses expressed in real terms do not exhibit great variation in time, the share of cost in GDP exhibits a decrease of economic burden in almost every risk factor; the overall indirect cost of all modifiable risk factors combined in 2000 was as much as 2.45% of GDP and it declined to 1.44% of GDP in 2017. The drop in share of GDP lost was highest in environmental and occupational factors (0.30% of GDP in 2000 to 0.16% in 2017), followed by metabolic factors (0.66% of GDP in 2000 to 0.36% in 2017) and behavioural factors (1.49% of GDP in 2000 to 0.92% in 2017). More than 0.2% of GDP lost in 2017 was estimated for deaths associated with alcohol use (0.44% of GDP), tobacco (0.33%), high systolic blood pressure (0.21%), and dietary risks (0.20%).

**Table 3:** Relative measures of production losses due to mortality attributable to modifiable risk factors in Poland in years 2000, 2010, and 2017

	Share of GDP (%)			Cost per death at working age^a^ (PLN)			Cost per capita^a^ (PLN)		
	2000 (%)	2010 (%)	2017 (%)	2000	2010	2017	2000	2010	2017
Environmental and occupational factors	0.303	0.192	0.156	256,765	230,149	303,252	91.60	78.86	80.47
Air pollution	0.242	0.145	0.105	277,891	253,776	329,768	73.29	59.63	54.45
Occupational risks	0.081	0.057	0.050	447,445	351,377	407,051	24.42	23.08	25.56
Other environmental risks	0.024	0.015	0.010	217,267	192,719	238,733	7.27	5.85	5.32
Unsafe water, sanitation, and handwashing	0.001	0.001	0.001	486,595	377,356	431,406	0.35	0.29	0.34
Non-optimal temperature	− 0.020	− 0.008	0.002	− 153,183	− 59,710	27,178	− 5.99	− 2.82	1.09
Behavioural factors	1.489	1.054	0.915	323,859	304,749	395,044	452.66	433.54	472.63
Alcohol use	0.566	0.487	0.443	455,417	418,786	540,676	172.83	200.48	229.77
Tobacco	0.643	0.394	0.325	243,237	218,210	277,097	195.36	161.83	166.78
Dietary risks	0.401	0.242	0.204	276,188	253,051	330,567	121.82	99.22	105.36
Drug use	0.046	0.041	0.039	529,330	474,719	626,374	14.05	17.03	20.14
Child and maternal malnutrition	0.100	0.049	0.030	801,258	761,739	899,136	29.62	20.00	15.64
Unsafe sex	0.042	0.024	0.020	354,627	341,666	491,551	12.76	9.98	10.44
Childhood sexual abuse and bullying	0.017	0.014	0.014	515,987	496,543	655,418	4.98	5.83	7.42
Low physical activity	0.012	0.007	0.007	231,129	212,121	287,402	3.53	3.06	3.90
Intimate partner violence	0.002	0.001	0.001	587,849	658,160	792,078	0.58	0.36	0.38
Metabolic factors	0.656	0.428	0.364	261,530	241,861	319,005	199.52	175.97	187.92
High systolic blood pressure	0.397	0.272	0.214	248,089	240,020	324,531	120.22	111.79	110.60
High body-mass index	0.326	0.219	0.195	271,134	250,541	331,138	99.69	90.30	101.04
High LDL cholesterol	0.309	0.169	0.133	281,180	258,439	338,112	93.92	69.73	68.93
High fasting plasma glucose	0.116	0.093	0.092	229,100	204,629	265,844	35.32	38.31	47.45
Kidney disfunction	0.072	0.041	0.032	277,743	249,909	327,185	21.69	16.93	16.55
Low bone mineral density	0.019	0.011	0.009	352,666	312,597	411,544	5.69	4.65	4.86
All risk factors	2.448	1.674	1.435	313,182	294,037	384,364	511.57	495.97	547.24

^a^Cost per death and per capita are expressed in real values (2017 is a base year)

The production loss caused by a single death due to all risk factors combined (Level 0) was 313.2 thousand PLN in 2000, 294.0 thousand PLN in 2010, and 384.4 thousand PLN in 2017. Deaths due to behavioural factors resulted in the highest average economic burden (395.0 thousand PLN per death in 2017), followed by deaths attributable to metabolic risks (319.0 thousand PLN) and environmental and occupational risks (303.3 thousand PLN). Per death cost of child and maternal malnutrition was by far the highest of all other risk factors; an average production loss due to this reason was 899.1 thousand PLN in 2017. The other factors with high per death costs were intimate partner violence (792.1 thousand PLN in 2017), childhood sexual abuse and bullying (655.4 thousand PLN), drug use (626.4 thousand PLN), and alcohol use (540.7 thousand PLN). All of these are behavioural factors and average loss per death is high here, because these risks lead to deaths at relatively young age generating greater losses.

Per capita production loss for all modifiable risk factors combined increased from 512 PLN in 2000 to 547 PLN in 2017. The magnitude of particular risk factors in this cost measure exhibits the same pattern as losses expressed as a share of GDP; therefore, the highest per capita burden was identified for behavioural factors (473 PLN in 2017), followed by metabolic factors (188 PLN) and environmental and occupational factors (80 PLN). Of single risk factors, the ones with highest per capita burden were alcohol use (230 PLN), tobacco (167 PLN), high systolic blood pressure (111 PLN), and dietary risks (105 PLN).

### Regional variation of production losses

The estimates of production losses attributable to all modifiable risk factors exhibit substantial regional variation; share of GDP lost in 2017 varied from 1.21% in podkarpackie region to 1.81% in łódzkie region. Cost per death was more than twice as low (265,172 PLN) in the region with the lowest per death loss (świętokrzyskie) compared to the wealthiest region where Poland’s capital city is located (mazowieckie; 612,724 PLN). The between-region difference was even more pronounced in per capita cost with 2.7-fold difference between regions at the top and bottom of the league table (Table 4).

**Table 4:** Regional variation of production losses due to mortality attributable to modifiable risk factors in Poland in year 2017

	Share of GDP (%)		Cost per death at working age^a^ (PLN)		Cost per capita^a^ (PLN)	
	Min (%)	Max (%)	Min	Max	Min	Max
Environmental and occupational factors	0.118^1^	0.199^2^	199,456^3^	460,433^4^	43.98^5^	116.67^4^
Air pollution	0.065^1^	0.140^2^	227,220^3^	512,170^4^	29.84^5^	81.48^4^
Occupational risks	0.043^4^	0.057^6^	272,422^7^	599,366^4^	17.51^8^	35.90^4^
Other environmental risks	0.008^4^	0.015^9^	170,026^7^	372,805^4^	3.75^8^	6.66^10^
Unsafe water, sanitation, and handwashing	< 0.001^7^	0.001^11^	309,568^7^	639,048^4^	0.16^7^	0.50^4^
Non-optimal temperature	− 0.016^3^	0.015^2^	− 235,865^5^	149,947^2^	− 5.93^5^	8.00^2^
Behavioural factors	0.742^8^	1.195^11^	271,878^7^	634,943^4^	267.68^8^	728.58^4^
Alcohol use	0.340^8^	0.643^11^	382,925^7^	863,869^4^	122.76^8^	372.43^4^
Tobacco	0.267^8^	0.392^11^	193,698^7^	433,611^4^	96.26^8^	238.65^4^
Dietary risks	0.172^1^	0.259^9^	228,428^3^	521,134^4^	64.14^5^	155.87^4^
Drug use	0.028^8^	0.050^11^	429,150^8^	1,029,473^4^	9.93^8^	31.73^4^
Child and maternal malnutrition	0.023^12^	0.036^13^	634,149^14^	1,416,713^4^	9.65^14^	23.86^4^
Unsafe sex	0.014^8^	0.028^10^	328,176^7^	788,963^4^	4.90^8^	16.21^10^
Childhood sexual abuse and bullying	0.006^9^	0.024^3^	459,164^3^	1,099,588^4^	2.50^9^	12.64^4^
Low physical activity	0.006^5^	0.009^9^	192,765^3^	449,355^4^	2.14^5^	6.27^4^
Intimate partner violence	< 0.001^8^	0.001^11^	551,047^3^	1,294,738^4^	0.16^8^	0.71^4^
Metabolic factors	0.306^1^	0.449^9^	221,344^3^	500,042^4^	116.26^8^	284.92^4^
High systolic blood pressure	0.176^1^	0.266^9^	225,688^3^	502,782^4^	69.82^5^	171.70^4^
High body-mass index	0.158^1^	0.243^9^	229,972^3^	518,351^4^	61.92^8^	157.46^4^
High LDL cholesterol	0.103^5^	0.176^9^	229,153^3^	526,755^4^	38.15^5^	104.31^4^
High fasting plasma glucose	0.070^8^	0.121^2^	180,367^3^	428,790^4^	25.22^8^	66.33^2^
Kidney disfunction	0.027^15^	0.040^9^	234,714^7^	510,429^4^	10.49^8^	24.31^4^
Low bone mineral density	0.008^1^	0.012^11^	290,804^14^	643,105^4^	3.09^8^	7.84^4^
All risk factors	1.205^8^	1.814^11^	265,172^7^	612,724^4^	315.70^8^	841.69^4^

^a^Cost per death and per capita are expressed in real values (2017 is a base year). Region’s labels: 1—pomorskie; 2—śląskie; 3—warmińsko-mazurskie; 4—mazowieckie; 5—podlaskie; 6—kujawsko-pomorskie; 7—świętokrzyskie; 8—podkarpackie; 9—opolskie; 10—dolnośląskie; 11—łódzkie; 12—małopolskie; 13—lubuskie; 14—lubelskie; 15—wielkopolskie

The lowest relative burden (share of GDP lost) of environmental and occupational factors was observed in pomorskie region (0.12% of GDP) which is characterized by the lowest air pollution in Poland [31]. On the other hand, the losses in the industrial and mining region of śląskie were almost twice as high (0.20% of GDP) and mortality due to air pollution therein was a single risk factor more burdening (0.14% of GDP) than all environmental and occupational factors in pomorskie. Considering behavioural factors, the lowest indirect cost of mortality was identified in podkarpackie (0.74% of GDP)—a region characterized by the lowest per person spending on alcohol and tobacco across all the regions in Poland [27]. This province experienced the lowest economic burden not only in alcohol use and tobacco risks, but also in drug use, unsafe sex and intimate partner violence. On the other hand, łódzkie region lost 1.20% of its GDP due to behavioural factors and was the most burdened in terms of mortality caused by alcohol use, tobacco, drug use, and intimate partner violence. There was less variation in relative production losses associated with metabolic factors with the share of GDP lost ranging from 0.31% in pomorskie to 0.45% in opolskie and these two provinces were at the top and bottom of the rankings for most of the metabolic factors.

Generally, for a vast majority of risk factors, both per death and per capita cost were the highest in mazowieckie region, which is not surprising as the GDP level therein is notably higher than in other regions of the country (160.5% of average per capita GDP in Poland in 2017 [27]). On the other hand, the lowest values in terms of per death cost were observed in the regions of warmińsko-mazurskie and świętokrzyskie while for per capita cost in podlaskie and podkarpackie. All these four provinces are located in eastern Poland and are characterized by low per capita GDP (69–72% of the average value) and this explains the low absolute burden.

### Sensitivity analysis

Sensitivity analysis exhibits a large variation in the estimates depending on risk factor and scenario applied with enormous changes in non-optimal temperature (this risk factor is described separately at the end of this sub-section). The assumption that all the deaths in particular age intervals occurred at the first (last) age of 5-year interval led to 22.6% higher (19.5% lower) losses for all modifiable risk factors combined than in a base scenario (BS) (scenarios Sc_1 and Sc_2; Table 5). With no discounting (Sc_3), the indirect cost was 65.9% higher than in the BS and it was 2.5- and 6-fold of the BS burden for unsafe water, sanitation, and handwashing; and child and maternal malnutrition, respectively. This great variation resulted from young age of those deaths due to these two risk factors. Applying discount rates of 2% (Sc_4) and 3.5% (Sc_5) led to 30.8% and 13.2% increase compared to BS in overall burden, respectively, with the highest variation in the same two risks as in Sc_3. The assumption of 0% (2%) fixed future economic growth rate (Sc_6 and Sc_7) resulted in lower deviations from BS of − 17.7% (− 5.1%) for all risk factors’ burden. Here, the pattern of relative changes for particular risk factors was similar to the one identified in discounting variation scenarios; unsafe water, sanitation and handwashing; child and maternal malnutrition; and intimate partner violence were those factors for which the cost estimates varied most notably. A ± 0.05 change in marginal productivity adjustment (Sc_8 and Sc_9) changed the cost by ± 7.7% in every risk factor, while the use of gross value added as a productivity measure decreased the estimates by 12.2% (Sc_11). Finally, if country-level input data were used instead of region-level figures, the results were hardly affected (Sc_10).

**Table 5:** Summary of sensitivity analysis scenarios for indirect cost of mortality attributable to modifiable risk factors in Poland in the year 2017

	Percentage deviation from base scenario (Table 2)										
	Sc_1 (%)	Sc_2 (%)	Sc_3 (%)	Sc_4 (%)	Sc_5 (%)	Sc_6 (%)	Sc_7 (%)	Sc_8 (%)	Sc_9 (%)	Sc_10 (%)	Sc_11 (%)
Environmental and occupational factors	29.6	− 24.6	48.9	23.7	10.4	− 14.7	− 4.3	− 7.7	7.7	0.3	− 12.2
Air pollution	27.0	− 22.9	56.6	26.6	11.5	− 15.7	− 4.5	− 7.7	7.7	− 0.2	− 12.2
Occupational risks	20.8	− 17.0	72.6	34.9	15.0	− 20.0	− 5.9	− 7.7	7.7	1.4	− 12.1
Other environmental risks	37.5	− 30.2	30.1	16.1	7.4	− 11.7	− 3.4	− 7.7	7.7	1.7	− 12.1
Unsafe water, sanitation, and handwashing	16.2	− 14.1	146.6	57.9	22.4	− 24.2	− 6.4	− 7.7	7.7	4.6	− 12.2
Non-optimal temperature	376.5	− 299.7	− 934.8	− 402.8	− 160.0	164.0	46.4	− 7.7	7.7	− 22.7	− 12.4
Behavioural factors	21.8	− 18.8	69.1	32.0	13.6	− 18.1	− 5.2	− 7.7	7.7	0.2	− 12.2
Alcohol use	15.7	− 14.3	66.9	32.7	14.3	− 19.6	− 5.8	− 7.7	7.7	− 0.2	− 12.2
Tobacco	32.6	− 27.1	36.1	18.8	8.6	− 13.1	− 3.9	− 7.7	7.7	0.9	− 12.2
Dietary risks	27.3	− 23.2	41.1	21.3	9.7	− 14.5	− 4.3	− 7.7	7.7	0.5	− 12.2
Drug use	13.1	− 12.3	74.1	35.9	15.6	− 21.0	− 6.2	− 7.7	7.7	− 0.5	− 12.2
Child and maternal malnutrition	− 5.3	5.4	516.6	182.3	64.8	− 53.5	− 11.8	− 7.7	7.7	0.6	− 12.2
Unsafe sex	17.1	− 17.4	55.7	28.1	12.5	− 17.8	− 5.3	− 7.7	7.7	0.5	− 12.2
Childhood sexual abuse and bullying	12.7	− 12.2	63.2	31.7	14.0	− 19.8	− 5.9	− 7.7	7.7	0.3	− 12.2
Low physical activity	32.1	− 27.1	35.3	18.6	8.5	− 13.0	− 3.9	− 7.7	7.7	− 2.4	− 12.2
Intimate partner violence	7.8	− 8.1	93.0	44.0	18.8	− 24.3	− 7.1	− 7.7	7.7	− 5.7	− 12.2
Metabolic factors	28.3	− 23.9	42.1	21.6	9.8	− 14.5	− 4.3	− 7.7	7.7	0.2	− 12.2
High systolic blood pressure	27.9	− 23.5	41.5	21.5	9.7	− 14.5	− 4.3	− 7.7	7.7	0.3	− 12.2
High body-mass index	27.4	− 23.6	41.1	21.3	9.6	− 14.4	− 4.3	− 7.7	7.7	− 0.1	− 12.2
High LDL cholesterol	27.0	− 23.1	39.3	20.6	9.4	− 14.2	− 4.2	− 7.7	7.7	0.0	− 12.2
High fasting plasma glucose	33.9	− 27.5	37.5	19.5	8.8	− 13.3	− 3.9	− 7.7	7.7	0.4	− 12.2
Kidney disfunction	27.0	− 22.7	53.8	26.0	11.3	− 15.8	− 4.6	− 7.7	7.7	1.2	− 12.2
Low bone mineral density	21.9	− 19.7	40.3	21.3	9.8	− 15.0	− 4.5	− 7.7	7.7	0.2	− 12.2
All risk factors	22.6	− 19.5	65.9	30.8	13.2	− 17.7	− 5.1	− 7.7	7.7	0.2	− 12.2

Scenarios: *Sc_1* all the deaths at the first age of 5-year interval, *Sc_2 *all the deaths at the last age of 5-year interval, *Sc_3* no discounting, *Sc_4* 2% discount rate, *Sc_5* 3.5% discount rate, *Sc_6* 0% future economic growth, *Sc_7* 2% future economic growth, *Sc_8* 0.6 marginal productivity adjustment, *Sc_9* 0.7 marginal productivity adjustment, *Sc_10* country-level data, *Sc_11* gross value added as a productivity measure

Extremally high variation in cost of deaths from non-optimal temperature results from the fact that for some age intervals this risk factor had a preventive effect on mortality (e.g., males aged 5–39 years) while for other groups it increased mortality (e.g., both sexes aged under 5 and > 40 years). This resulted in enormous high deviations from BS, particularly for those scenarios which assumed variation in the distribution of deaths across particular age intervals (Sc_1 and Sc_2) and those with a high sensitivity to time dimension (Sc_3—no discounting; Sc_4—2% discount rate; Sc_5—3.5% discount rate; Sc_6—0% future economic growth).

## Discussion

This is a first study that estimated production losses (indirect cost) associated with mortality attributable to a comprehensive set of modifiable health risk factors. Using regional data from Poland and 3 years, we assessed the economic burden of deaths due to all factors combined (Level 0), aggregated behavioural; environmental and occupational; and metabolic factors (Level 1) as well as twenty Level 2 factors including the most burdening modifiable risks such as tobacco, alcohol use, air pollution, and high BMI.

### Interpretation of estimates

The results show that modifiable risk factors combined led to losses (in real values) of 19.6 billion PLN in 2000 to 21.0 billion PLN in 2017. However, when expressing these values in relative terms, the burden declined from 2.45% of GDP in 2000 by 1 percentage point until 2017. This decline reflects relatively strong reduction of mortality at working age in Poland [32, 33]; for Level 0 risk factors, the YPPLL dropped from 1615 per 100,000 population in 2000 to 1207 in 2017 (− 25.3%). For environmental and occupational and metabolic risks, this drop was even more notable (> 1/3 in both). Only for two of the Level 2 factors, we observed an increase of YPPLL per 100,000 population in the 2000–2017 period, and these were drug use (+ 4.2%) and childhood sexual abuse and bullying (+ 7.9%).

Behavioural risks had a dominant role in mortality production losses, followed by metabolic; and environmental and occupational factors. Of twenty Level 2 risk factors, the ones that resulted in the highest losses were alcohol use, tobacco, high systolic blood pressure, and dietary risks. On the other hand, the cost per single death was the greatest for child and maternal malnutrition; intimate partner violence; childhood sexual abuse and bullying; and drug use—all of these being risks associated with deaths at young ages and, consequently, translating to substantial economic losses. Considering the dynamics of losses, it is noteworthy that for all twenty Level 2 risks, our estimates show the declining trend of relative burden (% of GDP lost). However, the dynamics of this trend was diversified; for tobacco, air pollution, dietary risks, unsafe sex, or high LDL cholesterol, the share of GDP lost halved, while in alcohol use, high fasting plasma glucose, drug use, or childhood sexual abuse and bullying, the improvement was modest. This time-trend diversity shows for which of the modifiable health risks the improvement has been achieved and points to those that should be tackled more efficiently to limit losses [34].

The interpretation of estimates for non-optimal temperature deserves closer inspection. For females, this factor had health deteriorating effect as expected for all other risks; however, in male mortality, we observed positive impact of non-optimal temperature meaning that the number of YPPLL was negative for this factor. This inferior relationship for females can be explained by the fact that the risk of death due to non-optimal temperature was identified as higher in females than in males in several studies (see, e.g., [35, 36]). However, this reasoning does not explain why do men experience health-enhancing effects of this risk factor. Moreover, the sex-specific trends of YPPLL show different time changes; the rate of years lost for women was stable across the period (3.3–3.5 YPPLL per 100,000 population in each of the years), while it changed substantially for men (− 23.7 YPPLL in 2000 and − 6.0 in 2017 YPPLL per 100,000 population) (see Supplementary file 1). Doubtlessly, the explanation behind these unexpected patterns might be of interest; yet, this study is not aimed at investigating such relationships.

Our findings exhibit the dominant role of male mortality in virtually all risks at each factor level. For all modifiable factors (Level 0) losses due to men deaths accounted for 83–85% of total cost. This clearly reflects greater male mortality at working age [37, 38] but also results from higher employment rates and longer labour market activity among men in Poland and these two factors strengthen the mortality effect [39]. The only risks associated with higher cost among women were those in which females are typically at higher risk of health damage, namely intimate partner violence and unsafe sex [40].

The results of regional analysis exhibit quite notable variation of production losses across Polish regions. For all modifiable risk factors analysed (Level 0), the relative burden was the highest in the region of łódzkie (1.81% GDP in 2017), which is a province characterized by the lowest life expectancy in the country [female (male) life expectancy—81.0 (72.5) years with average Poland values of 81.8 (74.1) years]. This region is characterized by the greatest burden across regions in numerous behavioural factors which had the greatest impact on losses, such as alcohol and tobacco use and this projects to its unfavourable position. Possibly, high mortality patterns and resulting economic burden therein might be associated with region’s capital (Łódź) progressing economic, demographic, and spatial degradation [41]. On the other hand, the south-eastern region of podkarpackie experienced the lowest losses of 1.21% GDP due to all modifiable factors. This region is characterized by the lowest frequencies of smoking among both sexes, the lowest obesity rates in women [42] and is one of three regions with the lowest emission of air pollutants, both in terms of gases and particulates emitted per 1 km^2^ [27]. All these factors contribute to low mortality and associated cost, but, on the other hand, the region is one of the least economically developed provinces. However, this study did not account for losses due to socio-economic factors. Possibly, the cost of deaths resultant from socio-economic deficiencies could be substantial for the regions of eastern Poland where economic measures deviate unfavourably from average country values. Further discussion of particular regions’ characteristics and their association with indirect cost borne are beyond the scope of this paper; yet, the region-specific figures might be of value for policy-makers.

Considering susceptibility of our estimates for changes in the model assumptions, the sensitivity analysis exhibits notable variation in some scenarios and for specific risk factors. As expected, no discounting resulted in high deviation from the base scenario (+ 65.9% for all risk factors combined), particularly for those risk factors in which death occurs at younger ages, with the extreme case of child and maternal malnutrition. Yet, the choice of discount rate or whether to apply discounting at all remains an unresolved issue [43, 44]; therefore, our results provide a set of scenarios to choose among in this respect. The other sensitivity scenarios resulting in meaningful changes from BS were those assuming different distribution of deaths in particular 5-year age intervals (Sc_1, Sc_2) and the assumption of no future economic growth (Sc_6). However, all these three scenarios seem to be implausible estimates and should rather be treated as extreme, potential values of losses experienced. Special caution is needed in interpreting sensitivity analysis results for non-optimal temperature where model assumptions play a critical role in the magnitude of losses estimated. The indirect cost for this risk factor varies extensively in some scenarios as explained in the results section.

### Comparison to other studies

To the best of our knowledge, this is the first study to estimate the production losses associated with an extensive set of mortality risk factors. Therefore, we cannot directly compare our figures with previous estimates. However, a recent American study aimed to assess health care spending attributable to risk factors classified in the same way as in our study [11]. According to this study, US health care spending attributable to modifiable risk factors was US$730.4 billion in 2016 and this corresponded to 27% of total spending on health care. High BMI had the highest attributable spending, followed by high systolic blood pressure, high fasting plasma glucose, dietary risks, and tobacco smoke [11]. This hierarchy shows that the three most notable factors in terms of treatment expenditure were metabolic risks. On the other hand, our findings show that the most burdening risks when it comes to mortality production losses were behavioural risks–alcohol use and tobacco. Yet, the other three factors generating high indirect cost in our study were high systolic blood pressure, dietary risks, and high BMI, and these overlap with US estimates of direct cost [11].

The other relevant studies for the comparison are ones using Canadian data to assess the cost of chronic disease risk factors (smoking, inactivity, overweight, and obesity) [45–47]. According to these studies, indirect cost of smoking accounts for slightly more than 1/3 of losses attributable to these four factors, while overweight, obesity, and inactivity contribute to these losses with shares of ~ 19%, ~ 27%, and ~ 19%, respectively. This cost structure differs from our estimates in which tobacco indirect cost is 43 times higher than for low physical activity (2017 estimate). Moreover, cost of obesity and overweight combined are higher than of smoking in Canadian studies while our estimates exhibit higher burden of tobacco compared to high BMI. These differences may arise from different cost components included in Canadian and our studies; the former encompass short- and long-term disability and mortality losses, while our study only accounts for mortality.

### Limitations of the study

Following caveats apply to our analysis. First, our estimates are prone to methodological shortcomings resulting from the design of GBD 2019 Study which was a source of input data for mortality estimates. As stated therein, estimates of risk-attributable burden for several risk factors (e.g., occupational risks, childhood sexual abuse, and intimate partner violence) are based on sparse evidence. Moreover, several important risk factors were not included in the GBD analysis and this includes social determinants of health such as educational attainment, poverty, and social exclusion—the factors increasingly recognized as being crucial for health outcomes. Additionally, in most cases, it was assumed that relative risks as a function of exposure were universal for all geographical and time settings [14]. Despite these data-driven challenges, GBD remains the most comprehensive study assessing the epidemiological burden of a range of modifiable risk factors and the figures provided therein are a recognized basis of policy actions. Second, the production losses estimation techniques itself suffer from methodological challenges, with the choice between HCM and friction cost approach being a major concern [4, 22, 24]. Our choice of HCM was based on the fact that it is used more frequently and seems to be more accepted approach to evaluate indirect costs [48]; moreover, friction period estimates for Poland are not available. Third, our estimates only report losses due to risk factors attributable mortality and no figures on morbidity cost are provided here. Still, we believe that our estimates provide a valuable insight into by-risk structure of economic burden as mortality and DALYs are highly correlated as stated above. Moreover, the studies assessing cost of mortality solely are numerous and our approach is not unique. Fourth, we used population-based data and this might bias the estimates in several ways. Particularly, the productivity or employment rates of those dying from particular risk factors might differ from average; e.g., alcohol-related mortality is potentially skewed towards those being less economically productive [49]. However, this risk of ecological fallacy is not unique for our study as only micro-level data control for socio-economic differences across subjects. Finally, our research only accounts for production losses in the formal sector. With this approach, the estimates exclude important parts of the productive economy such as volunteering, domestic duties, or childcare. This clearly translates to gender disparity in losses estimated with majority of cost identified in the male population. Consequently, inclusion of production cost borne in informal economy would plausibly result in higher share of burden assigned to females.

## Conclusion

In conclusion, the production losses attributable to all modifiable risk factors in Poland analysed within the GBD 2019 Study risk assessment framework decreased from 2.45% of GDP in 2000 to 1.44% in 2017. The risk factors having the greatest contribution to the burden of Polish economy were the behavioural ones with alcohol use and tobacco being major contributors. Moreover, dietary risks, high systolic blood pressure, and high BMI all generated substantial indirect cost. Importantly, this cost decreased notably with time for a majority of risk factors; this trend, however, was much slower for alcohol use which is the highest burdening single health risk nowadays. These results show that the public health policy towards ‘bads’ (alcohol and tobacco) shall be ranked high for the benefit of limiting not only health damage but also economic burden. Our findings provide economically hierarchised list of modifiable risk factors and as such are a contribution to inform policy-makers and public health advocates in prioritizing programmes to improve health and social well-being.

## Supplementary Information

Below is the link to the electronic supplementary material.

Supplementary file1 (DOCX 24 KB)

## Data Availability

We used publicly available data solely.
