# Supplementary material for: Production losses due to mortality associated with modifiable health risk factors in Poland
Source: Eur J Health Econ. 2021 Jul 8;23(1):33–45. doi: 10.1007/s10198-021-01345-6 (PMC8882090; doi:10.1007/s10198-021-01345-6)
Supplement: Supplementary file 1 — Supplementary file1 (DOCX 24 KB) [file 10198_2021_1345_MOESM1_ESM.docx]

Table S1. Years of potential productive life lost per 100,000 population attributable to modifiable risk factors in Poland in years 2000, 2010 and 2017

|  | 2000 |  |  | 2010 |  |  | 2017 |  |  |
| --- | --- | --- | --- | --- | --- | --- | --- | --- | --- |
|  | Males | Females | Total | Males | Females | Total | Males | Females | Total |
| **Environmental and occupational factors** | **228.7** | **57.6** | **286.3** | **169.2** | **38.7** | **207.9** | **142.2** | **29.9** | **172.2** |
| - Air pollution | 181.5 | 49.8 | 231.3 | 128.6 | 31.7 | 160.3 | 95.5 | 22.5 | 118.0 |
| - Occupational risks | 68.9 | 6.5 | 75.4 | 58.2 | 5.2 | 63.4 | 52.8 | 4.7 | 57.5 |
| - Other environmental risks | 19.8 | 2.4 | 22.2 | 13.4 | 1.7 | 15.1 | 9.9 | 1.4 | 11.2 |
| - Unsafe water sanitation and handwashing | 1.0 | 0.5 | 1.4 | 0.7 | 0.3 | 1.1 | 0.7 | 0.3 | 0.9 |
| - Sub-optimal temperature | -23.7 | 3.4 | -20.3 | -16.8 | 3.3 | -13.5 | -6.0 | 3.5 | -2.5 |
| **Behavioural factors** | **1,150.8** | **281.1** | **1,431.9** | **983.3** | **205.7** | **1,188.9** | **872.7** | **174.9** | **1,047.6** |
| - Alcohol use | 476.8 | 40.3 | 517.1 | 489.1 | 43.5 | 532.7 | 454.9 | 42.8 | 497.6 |
| - Tobacco | 476.9 | 120.1 | 596.9 | 339.7 | 77.9 | 417.6 | 285.4 | 65.1 | 350.5 |
| - Dietary risks | 304.6 | 64.3 | 368.9 | 215.3 | 40.4 | 255.7 | 186.5 | 34.4 | 220.9 |
| - Drug use | 36.7 | 5.7 | 42.4 | 39.9 | 5.6 | 45.5 | 38.2 | 6.0 | 44.2 |
| - Child and maternal malnutrition | 87.6 | 58.4 | 146.0 | 60.6 | 41.5 | 102.1 | 41.1 | 27.6 | 68.7 |
| - Unsafe sex | 4.2 | 39.6 | 43.8 | 4.4 | 24.3 | 28.7 | 3.5 | 20.6 | 24.1 |
| - Childhood sexual abuse and bullying | 14.1 | 0.5 | 14.6 | 14.6 | 0.7 | 15.3 | 15.1 | 0.7 | 15.8 |
| - Low physical activity | 7.9 | 2.9 | 10.8 | 5.8 | 2.0 | 7.9 | 6.1 | 1.9 | 8.1 |
| - Intimate partner violence | 0.0 | 2.1 | 2.1 | 0.0 | 1.1 | 1.1 | 0.0 | 0.9 | 0.9 |
| **Metabolic factors** | **492.4** | **116.3** | **608.7** | **378.1** | **77.5** | **455.6** | **331.1** | **64.0** | **395.0** |
| - High systolic blood pressure | 304.4 | 62.2 | 366.6 | 245.7 | 42.1 | 287.8 | 200.9 | 30.4 | 231.3 |
| - High body-mass index | 236.2 | 66.3 | 302.5 | 188.6 | 43.8 | 232.4 | 173.5 | 37.8 | 211.2 |
| - High LDL cholesterol | 244.9 | 36.8 | 281.7 | 156.9 | 20.3 | 177.2 | 126.7 | 15.8 | 142.5 |
| - High fasting plasma glucose | 85.0 | 23.5 | 108.5 | 80.9 | 18.4 | 99.4 | 82.6 | 17.1 | 99.7 |
| - Kidney disfunction | 51.1 | 17.1 | 68.3 | 35.6 | 10.1 | 45.7 | 28.5 | 7.5 | 36.1 |
| - Low bone mineral density | 13.9 | 2.9 | 16.7 | 10.0 | 1.7 | 11.8 | 8.6 | 1.5 | 10.1 |
| **All risk factors (Total)** | **1,284.6** | **330.7** | **1,615.3** | **1,107.4** | **245.1** | **1,352.6** | **996.5** | **210.3** | **1,206.8** |
